# Novel Genome-Editing Tools to Model and Correct Primary Immunodeficiencies

**DOI:** 10.3389/fimmu.2015.00250

**Published:** 2015-05-21

**Authors:** Lisa M. Ott de Bruin, Stefano Volpi, Kiran Musunuru

**Affiliations:** ^1^Division of Immunology, Boston Children’s Hospital, Harvard Medical School, Boston, MA, USA; ^2^Department of Pediatric Immunology, Wilhelmina Children’s Hospital, University Medical Center Utrecht, Utrecht, Netherlands; ^3^UO Pediatria 2, Istituto Giannina Gaslini, University of Genoa, Genoa, Italy; ^4^Division of Immunology and Allergy, Laboratory Center of Epalinges (CLE), University Hospital of Lausanne, Epalinges, Switzerland; ^5^Department of Stem Cell and Regenerative Biology, Harvard University, Cambridge, MA, USA

**Keywords:** Cas9, TALEN, ZFN, PID, SCID, endonuclease, nuclease, CRISPR/Cas9

## Abstract

Severe combined immunodeficiency (SCID) and other severe non-SCID primary immunodeficiencies (non-SCID PID) can be treated by allogeneic hematopoietic stem cell (HSC) transplantation, but when histocompatibility leukocyte antigen-matched donors are lacking, this can be a high-risk procedure. Correcting the patient’s own HSCs with gene therapy offers an attractive alternative. Gene therapies currently being used in clinical settings insert a functional copy of the entire gene by means of a viral vector. With this treatment, severe complications may result due to integration within oncogenes. A promising alternative is the use of endonucleases such as ZFNs, TALENs, and CRISPR/Cas9 to introduce a double-stranded break in the DNA and thus induce homology-directed repair. With these genome-editing tools a correct copy can be inserted in a precisely targeted “safe harbor.” They can also be used to correct pathogenic mutations *in situ* and to develop cellular or animal models needed to study the pathogenic effects of specific genetic defects found in immunodeficient patients. This review discusses the advantages and disadvantages of these endonucleases in gene correction and modeling with an emphasis on CRISPR/Cas9, which offers the most promise due to its efficacy and versatility.

## Introduction

Primary immunodeficiencies (PIDs) comprise a heterogeneous group of rare, chronic diseases in which part of the immune system is missing or functions improperly. PIDs are caused by a myriad of different genetic defects and their clinical manifestations may vary significantly. On the clinical spectrum of PID, severe combined immunodeficiency (SCID) is the most severe form of immunodeficiency. SCID is caused by many different genetic mutations that result in a developmental block in the production of T cells with an additional primary or secondary defect in B cells. NK cells may be lacking as well. SCID is characterized by increased susceptibility to life-threatening infections, particularly early in life. Newborn screening for SCID has been implemented in many states in USA, facilitating early detection and improving treatment outcomes (1–3). In addition to newborn screening, the advances in gene identification techniques, such as exome and genome sequencing, have greatly enhanced diagnostic capabilities in the field of PID. Over 230 PID-causing genes have been described and novel gene defects continue to be discovered (4). In parallel, the field of genome editing has progressed rapidly in the past few years, and many new tools are now available. These greatly ease the generation of *in vitro* models and animal models needed to study Mendelian disorders such as PID. Genome-editing techniques hold great promise for treatment by direct gene correction as well. This review will address these novel genome-editing methodologies and how these tools can be applied to model and correct PID.

### Hematopoietic stem cell transplantation

The current treatment of choice for SCID and other severe forms of PID is allogeneic hematopoietic stem cell transplantation (HSCT), which replaces defective hematopoietic lineages with functional cells. If a histocompatibility leukocyte antigen (HLA)-matched donor is available, conditioning chemotherapy is usually not indicated, because the patient has no T cells to cause rejection (5). However, HLA-matched donors may not be available. In those cases, depending on donor source and SCID genotype/phenotype, conditioning chemotherapy may be needed to facilitate robust and sustained engraftment of donor cells and improve immune reconstitution (6). Although results of HSCT have greatly improved over the years, when HLA-matched donors are lacking or when the recipients suffer from ongoing active infections or other serious complications, clinical outcomes are still suboptimal (5–12). This is due to risks of conditioning chemotherapy, graft rejection, graft-versus-host disease (GvHD), and delayed immune reconstitution. For these patients, gene therapy, in which gene-mutated autologous hematopoietic stem cells (HSCs) are complemented with a correct version of the gene, may offer an attractive alternative.

### Gene therapy using viral vectors

In order to complement autologous HSCs, CD34+ HSCs are harvested from the patient and then transduced with a viral vector containing a correct copy of the gene along with regulatory elements that control gene expression, such as promoters and enhancers. The viral vector allows integration of the therapeutic transgene into the HSC genome. HSCs transduced with the vector are then infused back into the patient. As in allogeneic HSTC, the number of successfully transduced HSCs required to obtain optimal reconstitution depends on the selective advantage of the corrected HSCs over the patient HSCs without the correct gene (13). In the first trials of gene therapy for PIDs, retroviral vectors were used in which expression of the normal transgene was driven by the retrovirus long terminal repeat (LTR). With this approach, successful and durable T cell reconstitution was achieved in patients with X-linked SCID [X-SCID (14, 15)], adenosine deaminase (ADA) deficiency (16–20), and Wiskott–Aldrich syndrome [WAS (21, 22)]. Unfortunately, several patients developed leukemia. These serious adverse events were caused by preferential integration of retroviral vectors in proximity of transcription initiation sites of genes (including oncogenes) and by the strong enhancer activity of the viral LTR, leading to increased and deregulated expression of the targeted oncogenes (23–26).

To counter these adverse effects, much effort has gone in the development of safer viral vectors. A gene therapy trial to correct X-SCID using a self-inactivating retroviral (SIN-RV) vector, in which the U3 enhancer was deleted from the LTR and expression was driven by the weaker eukaryotic human elongation factor 1α (EF1α) short promoter, is currently underway in Europe and in the USA. Preliminary results from this trial show a similar kinetics of T cell recovery compared to that of the previous trial, but with significantly less integration within proto-oncogenes. Long-term safety effects remain to be studied (27). In addition to the safer SIN-RV vectors, lentiviral vectors are promising. *In vitro* (28, 29) and *in vivo* studies (30–32) have demonstrated that lentiviral vectors integrate randomly in actively transcribed genes, without any preference for the transcription initiation sites and regulatory elements; thus, they are potentially safer (33). Therefore, several trials using SIN lentiviral (SIN-LV) vectors have been initiated (34), including a treatment trial of WAS that shows promising results (35).

### Genome editing using endonucleases

**Figure 1:**
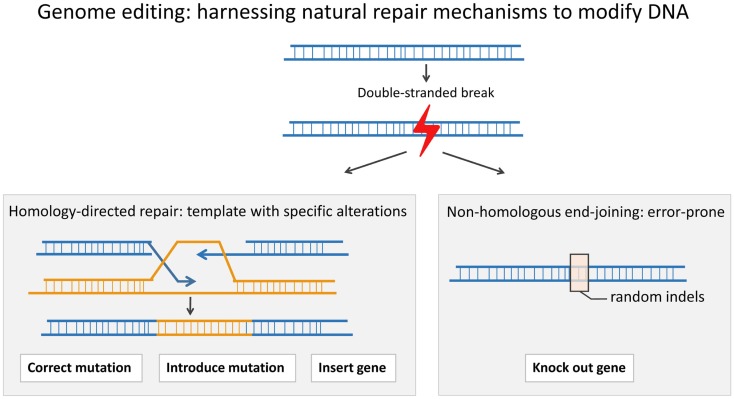
**A double-stranded break (DSB) in the DNA can be repaired through the process of homologous recombination (HDR) or through the error-prone process of non-homologous end-joining (NHEJ)**. In HDR, a template is used to correct the DSB. HDR can be used to precisely introduce a gene or part of a gene or even a point mutation, whereas NHEJ can result in insertions and/or deletions (indels) around the DSB. An indel can lead to a frameshift and an early stop codon.

While SIN-RV and SIN-LV vectors demonstrate a safer integration site profile, greater control over vector site integration is still desired. Engineered endonucleases that introduce double-stranded breaks (DSB) at specific sequences in the genomic DNA offer much more control over the integration site of viral vectors. Cells repair a DSB either through the error-prone process of non-homologous end-joining (NHEJ) or through homology-directed repair (HDR) in which a highly homologous template, either a sister chromatid or an exogenous double-stranded or single-stranded DNA template, is copied accurately. HDR can be used to either insert a gene into a specific “safe harbor” or to replace a defective gene *in situ* (Figure 1). “Safe harbors” are regions in genomic DNA that do not contain oncogenes and that can be disrupted without adverse consequences. One such safe harbor is the adeno-associated virus integration site 1 (*AAVS1*) locus. In order to insert a functional copy of the PID-causing gene into a specific locus, such as *AAVS1*, one can use an engineered endonuclease to introduce a DSB at the site and a DNA repair template containing the gene flanked by two homology arms that match the *AAVS1* sequence (36–39).

Alternatively, instead of adding a normal copy of the gene in the “safe harbor,” one can correct the defective PID gene *in situ*. In this case, the DSB is introduced close to the mutation, and then, a repair template, containing the correct sequence flanked by two homology arms matching the sequences surrounding the target site, is inserted. When correcting the actual mutation itself, the endogenous promoter, enhancer, and other regulatory elements are used, and thus, physiological gene expression is preserved. This is beneficial when aiming to correct tightly regulated genes, such as *Recombination-activation gene 1 (RAG1)* and *Recombination-activation gene 2 (RAG2)*, the genes required for VDJ recombination during T cell and B cell development (40). In addition, *in situ* correction is ideal for dominant-negative mutations; in these cases, simple addition of the normal gene would be inadequate to rescue the phenotype, and a specific correction of the mutation is required. The endonucleases can be designed to only target the mutant sequence and spare the wild-type sequence. For the majority of PIDs, the disease phenotype is caused by recessive mutations. In these cases, correcting one allele is sufficient to rescue the phenotype.

When using engineered endonucleases, several aspects need to be considered. First, the efficiency of introducing DSBs at the target site, the on-target efficiency, is important. When testing the endonuclease, on-target efficiency can be inferred from the proportion of alleles in a batch of cells showing deletions or insertions (indels) at the target site, because some of the introduced DSBs were repaired by the error-prone process of NHEJ. These indels can be easily captured using next-generation sequencing or alternatively, by studying the heteroduplex DNA hybridization of PCR products from the target site (e.g., with the Surveyor^®^ assay). Second, a common concern with the use of endonucleases is off-target mutagenesis. This is the inadvertent introduction of mutations caused by DSBs at genomic sites other than the target site. Currently, several sequencing techniques are available to check for off-target mutagenesis. Some will be addressed in the next section when discussing different endonucleases.

A third aspect that needs to be considered when using engineered endonucleases is how to deliver the endonuclease and the DNA template to the cell. For *ex vivo* therapeutic applications, such as gene targeting in HSCs harvested from a patient, nucleofection is a non-viral method to introduce polynucleotides into the cells (41). Its disadvantages include toxicity to the cells and low efficiency. Alternatively, viral vectors that do not integrate in the genome, such as integrase-deficient lentiviral vectors, adenoviral vectors, and vectors based on adeno-associated viruses can be used. These viruses enter the cell and express the endonuclease, without inserting it into the genome. These have been proven effective tools to deliver both the repair constructs and the endonucleases (42–45). *In vivo* therapeutic applications present a greater challenge in that the delivery method must efficiently and specifically target the desired cells and spare the other cells within the whole body.

## ZFNs, TALENs, and CRISPR/Cas9

**Figure 2:**
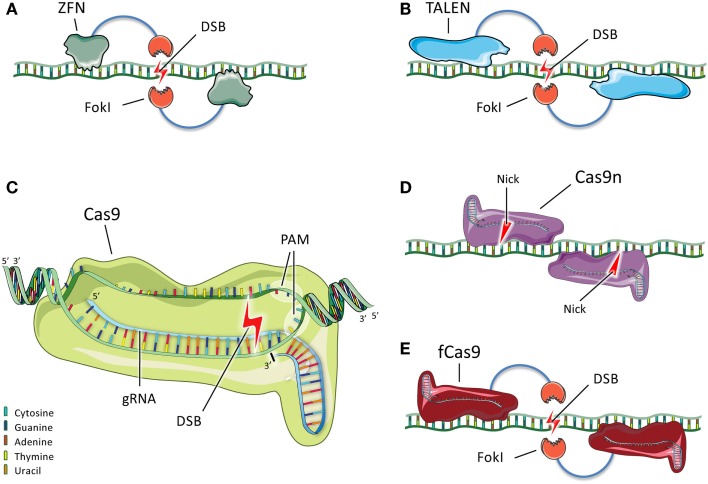
**Schematic representation of ZFNs, TALENs, and CRISPR/Cas9**. **(A)** Two ZFN dimers bind DNA and position their *Fok*I nuclease domains such that they dimerize and generate a double-stranded break (DSB) between the binding sites. **(B)** TALENs, like ZFNs, bind DNA and generate a DSB upon dimerization of their *Fok*I domains. **(C)** In the most commonly used CRISPR/Cas9 system, Cas9 forms a complex with a gRNA that recognizes and hybridizes a 20-bp protospacer in the genome. Cas9 binds the adjacent PAM sequence and introduces a DSB 3 bp upstream of the PAM sequence. **(D)** Cas9 nickases (Cas9n) are mutant variants that bind to flanking DNA sequences and generate single-strand nicks instead of DSBs. Two nicks are the equivalent of a DSB. **(E)** Another variant consists of catalytically inactive Cas9 (fCas9) fused to a *Fok*I nuclease domain. When two *Fok*I nucleases dimerize because the Cas9 proteins bind to flanking DNA sequences, a DSB is introduced between the binding sites.

Although a number of different genome-editing technologies are now in use, we describe three types of engineered endonucleases that have found broad use in the biomedical community, with a particular focus on the most recently developed nuclease system, CRISPR/Cas9, which has attracted widespread attention for its efficacy and versatility (Figure 2).

### Zinc finger nucleases

Zinc finger nucleases (ZFNs) consist of a pair of endonuclease domains of the bacterial *Fok*I restriction enzyme flanked by two site-specific DNA-binding domains (Figure 2A). Upon binding of these domains, the *Fok*I domains dimerize and introduce a DSB (46). Even though academic consortia have developed open-source libraries for ZFN construction (42, 47), engineering of site-specific ZFNs remains difficult for non-specialists. Depending on the ZFN architecture used, only limited sites in a genomic region can be targeted, which might be problematic if a specific mutation needs to be corrected (43–45, 48). One study showed off-target mutagenesis with ZFNs in one out of ten sites with a sequence similar to the target site (37). Two other studies also found that DSBs were introduced in off-target sites using ZFNs in a human tumor cell line (49, 50). One of the ZFNs tested was designed to correct mutations causing X-SCID, i.e., mutations in the gene encoding the Interleukin 2 receptor gamma (*IL2RG*) (49, 51). Variants of the endonucleases have been developed to reduce off-target mutagenesis. These consist of a mix of two distinct ZFNs with different *Fok*I domains that are obligate heterodimers, such that a ZFN pair introduces a DSB only when the two distinct ZFNs are able to bind adjacent DNA regions (52–54).

### Transcription activator-like effector nucleases

Transcription activator-like (TAL) domains are tandem arrays with 10–30 repeats, each 33–35 amino acids long that bind and recognize extended DNA sequences (55). One domain of the TAL repeats is fused to a *Fok*I endonuclease domain, creating a TAL effector nuclease (TALEN), similar to the ZFN. Upon binding of the two TALENs to flanking DNA sequences, the *Fok*I domains dimerize and introduce a DSB at the target site (Figure 2B). TALENs are easier to design and have fewer constraints on site selection than ZFNs. A disadvantage of using TALENs is their size. The DNA sequence encoding each TALEN is more than 3 kb in size, compared to only 1 kb for each ZFN. The larger size makes it harder to deliver TALEN-expressing plasmids into cells. Moreover, the highly repetitive nature of TALEN sequences due to their tandem arrays makes them more challenging to package into viral vectors for delivery (56). With respect to off-target mutagenesis, two studies showed this phenomenon was minimal with TALENs (57, 58). Unfortunately, not many studies have compared TALENs and ZFNs directly. However, one study suggested that when targeting the *CCR5* gene, less off-target mutagenesis was seen with TALENs than with ZFNs (59).

### CRISPR/Cas9

The latest in a series of new genome-editing tools is the clustered regularly interspaced short palindromic repeats (CRISPR)/CRISPR associated 9 (Cas9) system. Cas9 is a protein used by bacteria to destroy foreign DNA. The foreign DNA is cleaved and incorporated as small sequences – called protospacer sequences – into the bacterial genome, and these sequences are then transcribed as short CRISPR RNAs (crRNAs). These crRNAs are then used to target and destroy any foreign DNA sequences that enter the cell and match those sequences. The bacterial Cas9 nuclease and a crRNA form a ternary complex with a second RNA component, the trans-activating crRNA (tracrRNA), which has a fixed sequence. This complex can engage double-stranded DNA, with the crRNA hybridizing the protospacer sequence and Cas9 binding a specific protospacer-adjacent motif (PAM). Once the complex is engaged, Cas9 introduces a DSB 3 bp upstream of the PAM (60). After the characterization of the CRISPR/Cas9 system in bacteria, investigators found that it could be used to introduce DSBs efficiently in mammalian DNA. The RNA components of the CRISPR/Cas9 system can be separate crRNA and tracrRNA molecules, or the two RNA molecules can be combined into a single guide RNA [gRNA (61–64)]. The Cas9 protein from the species *Streptococcus pyogenes* is the most commonly used at present and uses a PAM with the sequence NGG. When Cas9 is used with a single gRNA (as is now usually the case), CRISPR/Cas9 represents a simple two-component system (Figure 2C).

The advantages of CRISPR/Cas9 are the high efficiency of introducing DSBs into the genomes of mammalian cells and the ease of engineering. The Cas9 nuclease is always the same; to target a different region of the genome, only the protospacer region (20 nucleotides) of the gRNA needs to be altered. While theoretically the PAM requirement could be a limitation, on average the NGG PAM sequence needed by *S. pyogenes* Cas9 can be found every 8 bp in the genome, making it very likely to find a CRISPR/Cas9 target site near the mutation that needs to be corrected (63). In contrast, the PAM sequence used by *Streptococcus thermophilus* Cas9, NNAGAAW, occurs on average every 64 bp; the *Neisseria meningitidis* Cas9 protein requires a NNNNGATT PAM, which occurs on average every 128 bp (63, 65, 66).

As with other engineered nucleases, the main concern regarding the use of CRISPR/Cas9 is off-target mutagenesis. Such mutagenesis appears to be highly gRNA dependent and most often occurs at sites with sequence similarity to the protospacer, with up to several mismatches tolerated; more mismatches are tolerated as the distance from the PAM increases (61, 62, 67–70). The number of potential off-target binding sites of Cas9 can vary widely, depending on the gRNA used (71, 72).

Moreover, some studies suggest that even when the CRISPR/Cas9 is able to bind to an off-target site, the mismatches prevent actual DNA cleavage (45, 48). A novel method to systematically assess off-target mutagenesis, the so-called genome-wide, unbiased identification of DSBs, enabled by sequencing (GUIDE-seq), was recently reported (73). This method is based on the detection of small synthetic double-stranded DNA oligodeoxynucleotides that are incorporated in the genomic DNA at the site of the DSBs through NHEJ.

To reduce off-target mutagenesis, many different variants of the CRISPR/Cas9 system with higher target specificity are being developed. In one strategy, the length of the gRNA protospacer is reduced by up to three nucleotides, which appears to make the gRNA less tolerant of mismatches and less likely to bind to off-target sites, thereby reducing the rate of off-target mutagenesis (74). A different approach uses Cas9 “nickases,” which are mutated variants of Cas9 that each introduce a single-stranded break (called a nick) instead of a DSB (Figure 2D). When two distinct gRNAs matching to two distinct sequences flanking the target site are used, Cas9 will produce two separate nicks that together are the equivalent of a DSB and can thus induce repair by NHEJ or HDR. Off-target mutagenesis is substantially reduced with the double-nickase strategy because the two nicks occur in proximity only when Cas9 binds to two adjacent sequences that resemble the protospacers, which is very unlikely to occur elsewhere in the genome (75–77). Finally, investigators have combined the most desirable properties of CRISPR/Cas9 and ZFNs/TALENs by fusing a catalytically dead Cas9 to a *Fok*I domain. Two Cas9-*Fok*I fusion proteins are guided to flanking sequences around a target site by a pair of gRNAs. Upon DNA binding of the Cas9 domains, the *Fok*I domains dimerize and generate a DSB (Figure 2E). As with the double-nickase strategy, binding of Cas9 to two separate nearby sequences is required for the generation of a DSB, and accordingly the off-target mutagenesis rate is greatly reduced (78, 79).

## Genome Editing Results in Human Stem Cells

A number of studies have demonstrated the feasibility of genome editing in human stem cells with ZFNs, TALENs, and CRISPR/Cas9 (36, 37, 51, 62, 77, 80–84). In one such study, intestinal stem cells of cystic fibrosis patients with homozygous delta508 mutations in the *CFTR* gene were corrected using CRISPR/Cas9. Corrected and uncorrected stem cells were differentiated into organoids and compared. The corrected organoids showed swelling in response to forskolin treatment, as expected in the presence of a functional CFTR protein, whereas the mutant organoids failed to swell (85). Another group transfected human HSCs with ZFNs that disrupted *CCR5*, the chemokine receptor used by human immunodeficiency virus (HIV) to infect cells. The targeted cells were transplanted into immunodeficient NOD/SCID/IL2rγ-null (NSG) mice, which then exhibited human hematopoiesis. Moreover, when the transplanted mice were infected with HIV, there was a selective survival advantage for the *CCR5* knockout cells, protection of the human-derived T cell populations, and a reduction in HIV viral load (41).

In SCID and most types of PID, multiple cell types of the immune system are defective. In order to restore the immune function one needs to target the patient’s own HSCs (84). The most common form of SCID, X-SCID, is caused by mutations in the gene encoding interleukin 2 receptor gamma (*IL2RG*). Several groups have successfully used ZFNs to target and induce HDR in the *IL2RG* locus in various human cell types, including HSCs and embryonic stem cells [ESCs (51, 84, 86)]. One of these studies corrected an *IL2RG* mutational hotspot in HSCs using ZFNs and showed multilineage hematopoietic differentiation upon transplantation of gene-corrected cells into NSG mice (84). Another study successfully used specific TALENs to target and induce HDR in the *IL2RG* locus of Jurkat cells (87).

Challenges when targeting HSCs are the low efficiency of HDR and the risk of losing multilineage potential when manipulating and expanding gene-corrected cells *in vitro*. Several cell types, such as T cells, HSCs, and fibroblasts, can be reprogramed into induced pluripotent stem cells (iPSCs) by transducing these cells with a SIN-lentiviral vector expressing three pluripotency genes, *OCT4*, *SOX2*, and *KLF4* (88–90). Studies have shown the feasibility of iPSC gene targeting to correct hematopoietic diseases, such as sickle cell disease *in vitro* using ZFNs (82, 91) or TALENs (92). Many different iPSCs from a number of patients with distinct immunodeficiencies have now been generated (38, 39, 93, 94). These patient iPSCs can be corrected, or alternatively, an original patient somatic cell can be corrected before being reprogramed into iPSCs. ZFNs have been used to correct chronic granulomatous disease (CGD) by introducing up to five different functional genes into the *AAVS1* safe harbor in iPSCs generated from peripheral HSCs. Using *in vitro* myeloid differentiation, normal granulocytes were generated from the corrected iPSCs (38, 39). Successful differentiation of human iPSCs into T cells *in vitro* has been recently reported (95–97), making it possible to test the ability of genome editing to restore T cell differentiation capacity of iPSCs from patients with SCID. However, *in vivo* use of gene-edited iPSCs for correction of human PIDs is not yet ready for the clinic because of safety concerns related to the tumorigenic potential of iPSCs and because of difficulty in generating definitive HSCs from human iPSCs. Nonetheless, these recent achievements with *in vitro* targeted differentiation of human iPSCs have great value in that they enable preclinical efficacy and safety studies of genome-editing approaches that may eventually be applied to human HSCs.

## Modeling PIDs Using Genome Editing

### *In vitro* models

Even though gene targeting using engineered endonucleases is not ready to be applied in a clinical setting, it already offers a valuable tool to model diseases at the cellular level. CRISPR/Cas9 has been shown to be particularly efficient in the generation of knockout cell lines; as described above, after a DSB has been introduced into the genomic region matching the protospacer sequence of the gRNA, the cell uses either HDR or NHEJ to repair the defect. NHEJ can result in indels, which in turn can cause frameshifts and the occurrence of premature stop codons. A knockout cell generated in this way can be clonally expanded into a cell line that can be used for modeling studies. In addition, a useful property of the CRISPR/Cas9 system is that multiple genes can be knocked out simultaneously if several gRNAs are used together (multiplexing). A particular advantage compared to RNA interference is that CRISPR/Cas9 can be used to target regions in the non-coding genome [e.g., promoter and enhancer regions (98–101)].

**Figure 3:**
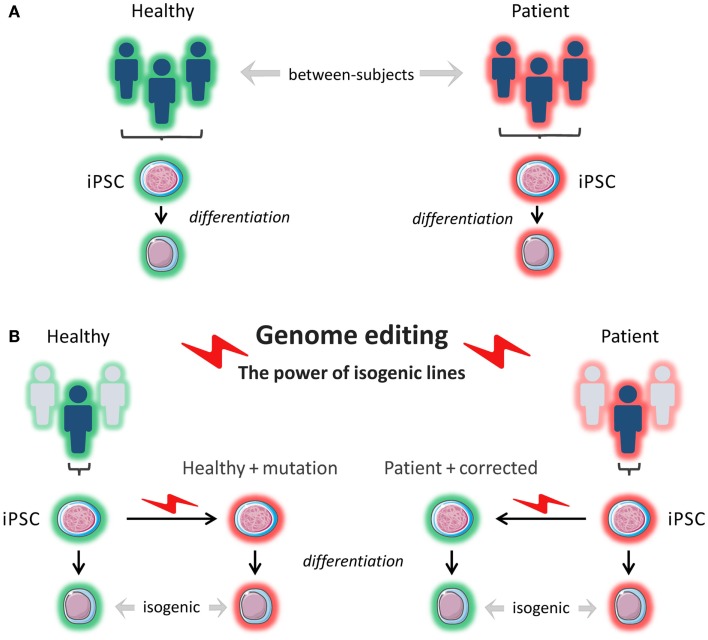
***In vitro* modeling**. **(A)** Induced pluripotent stem cells (iPSCs) are reprogrammed from a patient(s) and from a healthy control(s). The iPSCs are differentiated into a cell type of interest, and the phenotypes of the patient-derived cells are compared to the phenotypes of the healthy control cells. The cells that are compared do not have the exact same genetic background (genetically and epigenetically unmatched). This can lead to confounding. **(B)** Using genome editing with engineered nucleases like ZFNs, TALENs, and CRISPR/Cas9, a pathogenetic mutation can be corrected in patient-derived cells or introduced into healthy control cells, and isogenic cell lines (i.e., identical genetic background) can be compared for relevant phenotypes.

The advent of next-generation sequencing has stimulated a new wave of discovery of novel inborn errors of immunity (102). The ability to correct patient-specific iPSCs, or conversely, to introduce patient-specific mutations into a wild-type iPSC line using endonucleases represents an invaluable tool to prove the pathogenicity of newly discovered mutations and to gain insight into disease mechanisms in different cell types, depending on patients’ phenotypes. This approach also makes it possible to study the contribution of genetic background to the phenotypes arising from specific mutations by comparing patient-derived iPSCs with wild-type iPSCs into which the same mutations are introduced (Figure 3). In a recent study, iPSCs were generated from patients with Parkinson disease caused by the G2019S mutation of the LRRK2 gene and from healthy controls. When comparing the whole-genome gene expression patterns, the investigators found a high degree of heterogeneity among the different iPSCs lines. However, when they used ZFNs to correct the mutation in three of the patient-derived iPSC lines and compared these lines to the original lines, and when they introduced the mutation into a healthy control line and compared this line to the original line, the lines were much more closely matched with respect to gene expression (83). This shows the importance of comparing isogenic lines, as confounding due to differences in genetic background is minimized.

### Animal models

Traditionally, animal models have been generated using homologous recombination: embryonic stem cells (ESCs) are electroporated with a highly homologous DNA template containing the sequence to be inserted but without using an engineered endonuclease to introduce a DSB. This approach results in a very low efficiency and requires the inclusion of an antibiotic resistance gene in the inserted sequence for the selection of cells in which HDR has occurred. ESCs with the desired inserted sequence are then expanded, injected in blastocysts, and subsequently implanted in pseudogestant females. The resulting chimeric animals have to be further bred until the introduced mutation is transmitted through the germline. With the currently available genome-editing tools, this process can be greatly streamlined. Via the introduction of a DSB at the desired target site, a specific sequence can be efficiently introduced into ESCs without the need for an antibiotic resistance gene. Furthermore, to create a gene knockout, one can simply rely on NHEJ to produce indels leading to frameshifts and early stop codons.

**Figure 4:**
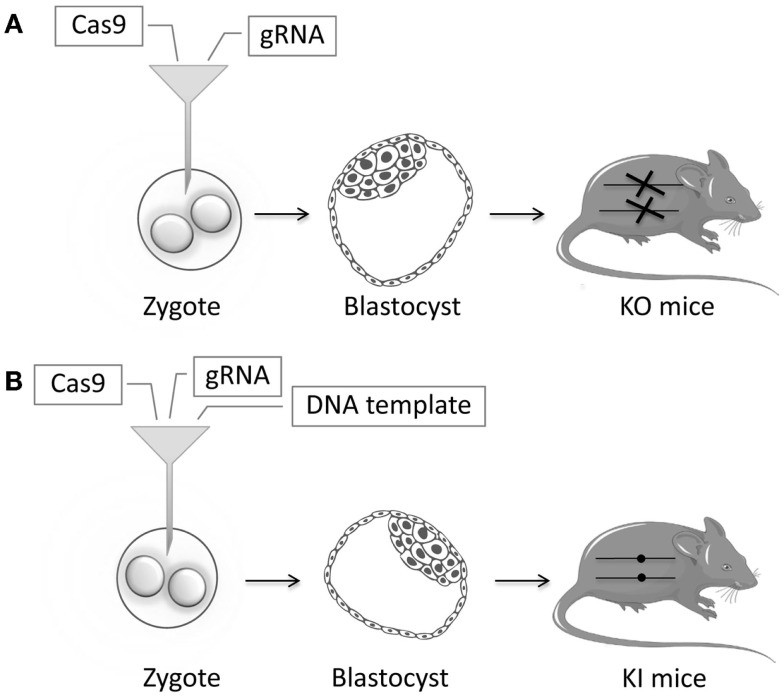
**Schematic representation of zygote injection with CRISPR/Cas9**. **(A)** Injection of gRNA and Cas9 will lead to indels that can lead to a frameshift and an early stop codon thereby creating Knockout (KO) mice. **(B)** Addition of a highly homologous DNA template containing a specific mutation will result in Knock-in (KI) mice, through the process of homology-directed repair (HDR). Reagents are injected in the cytoplasm of the zygote. Alternatively, these can be injected in the pronucleus of the zygote, but cytoplasmic microinjection is simpler and less toxic.

In recent years, many animal models have been successfully generated using ZFNs, TALENs, or CRISPR/Cas9. TALENs and CRISPR/Cas9 have been used to generate knockout *Caenorhabditis elegans* models by injecting the endonucleases into the gonads (103–105). Similarly, more complicated animal models can be generated by injecting the endonucleases in mRNA form directly in zygotes (Figure 4). In the case of CRISPR/Cas9, this means that both the Cas9 and gRNA in RNA form are injected. Knock-in models can be generated by adding a DNA template to the injection mix, usually in the form of a single-stranded DNA oligonucleotide. Zebrafish models have been generated by injecting ZFNs or TALENs or CRISPR/Cas9 directly into the zygote (106–109). This has been done in murine zygotes with ZFNs (110–112), TALENs (113, 114), and extremely efficiently with CRISPR/Cas9 (115–117). New mouse models can be generated in just a few weeks, instead of taking 1–2 years as in the conventional strategy. With CRISPR/Cas9, the specific gRNA needed for the injections can be generated in a simple one-day procedure (118). NSG mice have been efficiently generated in this way (119). In other studies, the IgM locus has been successfully knocked out in rats via the injection of ZFNs and TALENs directly into the zygotes (120, 121). Similarly, a rat model of X-SCID has been generated using ZFNs (122). The multiplexing capacity of CRISPR/Cas9 has allowed for multiple genes being knocked out simultaneously (123). Endonucleases have been used to generate knockout models in animals not previously amenable to efficient genetic modification: rabbits with *IL2RG*, *RAG1*, or *RAG2* knockout (124–127); hamsters with *STAT2* knockout (128); mutant pigs (129–131); and most impressively, monkeys with *RAG1* knockout (132). These kinds of animal models will enable disease studies of unprecedented sophistication.

## Conclusion

During the last few years, the field of genome editing has shown tremendous progress. Currently, several endonucleases are available. We have described their advantages and disadvantages and how they can be used to model and correct PIDs. Efficiency and ease will continue to improve with further refinement of these tools, while endonuclease variants with increased specificity are being actively developed. To use genome editing in a clinical setting for the treatment of PID will require a further reduction in off-target mutagenesis and an improved yield of gene-corrected HSCs so that a sufficient number of cells for autologous transplantation and engraftment can be obtained. Despite of these issues, we expect the impact of genome editing on modern medicine to be revolutionary.

## Conflict of Interest Statement

The authors declare that the research was conducted in the absence of any commercial or financial relationships that could be construed as a potential conflict of interest.
